# Supplementary material for: A Systematic Review to Define the Multi-Faceted Role of Lysine Methyltransferase SETD7 in Cancer
Source: Cancers (Basel). 2022 Mar 10;14(6):1414. doi: 10.3390/cancers14061414 (PMC8946661; doi:10.3390/cancers14061414)
Supplement: Supplementary file 1 [file cancers-14-01414-s001.zip › supplementary figures_final.pdf]

Review

A Systematic Review to Define the Multi-Faceted Role of the Lysine Methyltransferase SETD7 in Cancer

Fátima Liliana Monteiro [1], Cecilia Williams [2] and Luisa A. Helguero [1,*]

1 Institute of Biomedicine – iBiMED, Department of Medical Sciences, University of Aveiro, Portugal;
2 SciLifeLab, Department of Protein Science, KTH Royal Institute of Technology, Sweden;
* Correspondence: luisa.helguero@ua.pt

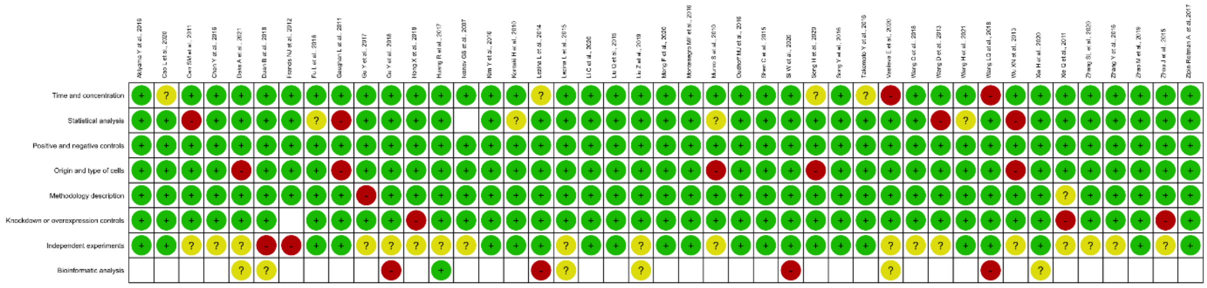

Supplementary Figure S1 Risk of bias in individual studies. The following criteria were considered for functional studies: information on time and concentration when a treatment (like chemicals, knockdown, etc.), description and complete report of statistical analysis, usage of positive and negative controls, description of the origin and type of cells used, methodology detail, assessment of gene/protein expression after loss and/or gain of function experiments and usage of independent experiments, and description of bioinformatics methodology used for analysis of public datasets or own cohorts. Green: low risk of bias; yellow: risk of bias is unclear in at least one domain, but no domains with high risk; red: high risk of bias; white: not applicable to the study in question.

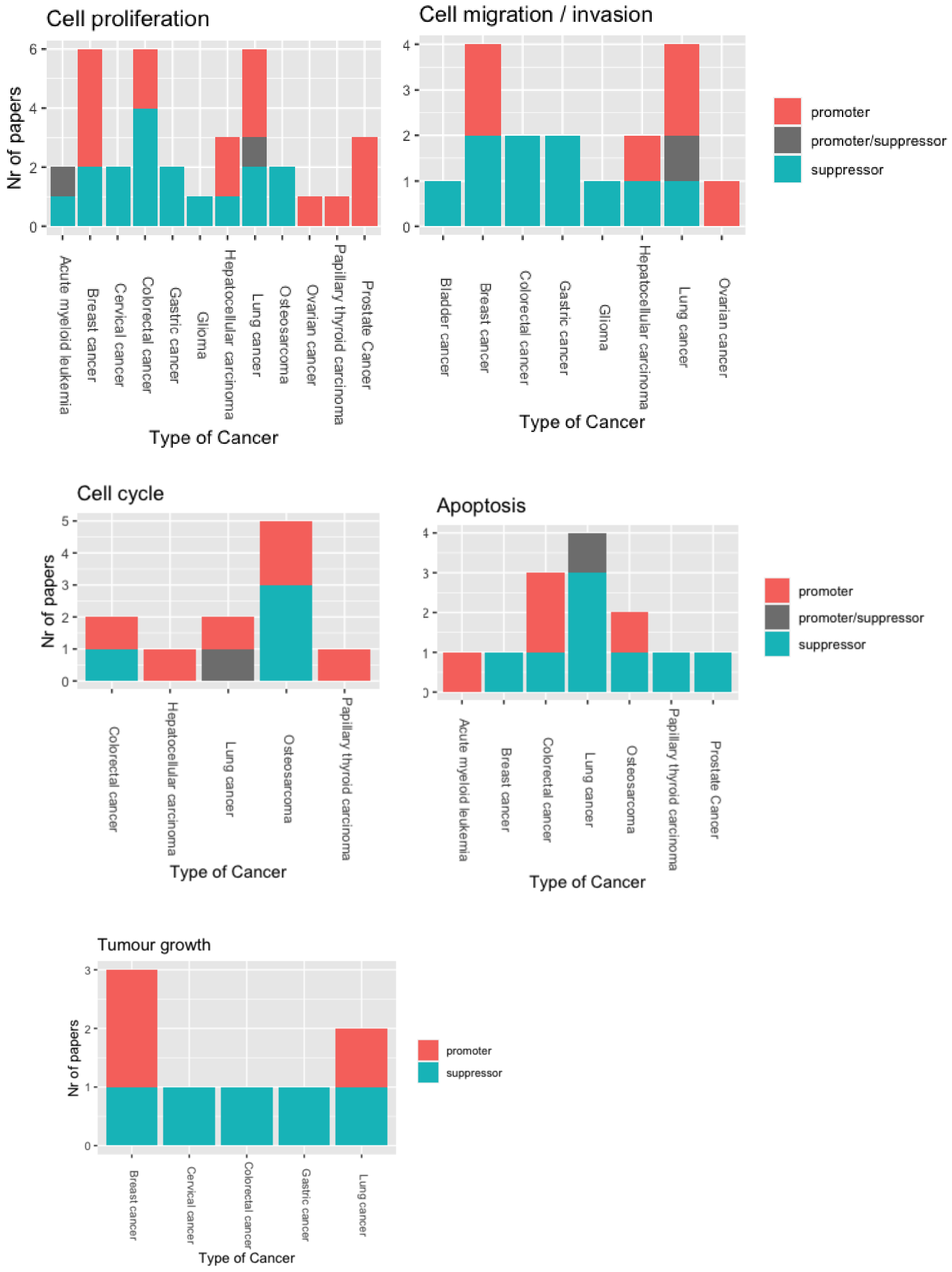
Cell proliferation
Nr of papers
6
4
2
0
Acute myeloid leukemia
Breast cancer
Cervical cancer
Colorectal cancer
Gastric cancer
Glioma
Hepatocellular carcinoma
Lung cancer
Osteosarcoma
Ovarian cancer
Papillary thyroid carcinoma
Prostate Cancer
Type of Cancer
Cell migration / invasion
4
3
2
1
0
Bladder cancer
Breast cancer
Colorectal cancer
Gastric cancer
Glioma
Hepatocellular carcinoma
Lung cancer
Ovarian cancer
Type of Cancer
promoter
promoter/suppressor
suppressor
Cell cycle
Nr of papers
5
4
3
2
1
0
Colorectal cancer
Hepatocellular carcinoma
Lung cancer
Osteosarcoma
Papillary thyroid carcinoma
Type of Cancer
Apoptosis
4
3
2
1
Acute myeloid leukemia
Breast cancer
Colorectal cancer
Lung cancer
Osteosarcoma
Papillary thyroid carcinoma
Prostate Cancer
Type of Cancer
promoter
promoter/suppressor
suppressor
Tumour growth
Nr of papers
3
2
1
0
Breast cancer
Cervical cancer
Colorectal cancer
Gastric cancer
Lung cancer
Type of Cancer
promoter
suppressor

(a)

(**b**)

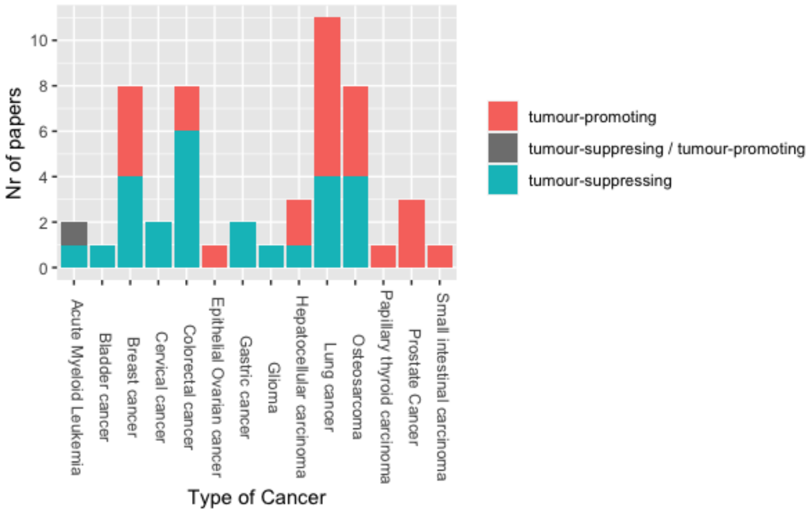

(**c**)

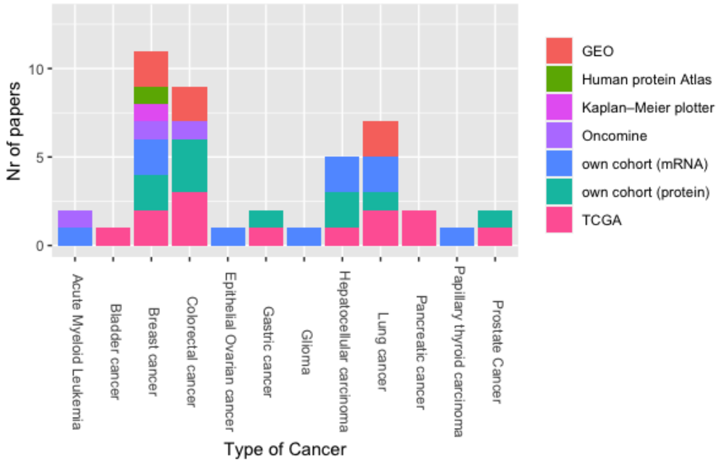

Supplementary Figure S2 Analysis of the literature. (**a**) *Literature categorization according to SETD7 function over the most studied cancer-related processes by cancer type.* For the most studied cancer-related cellular processes (cell proliferation and cell migration / invasion), studies were grouped by cancer type and SETD7 was categorized as having a promoter (red) or suppressor (green) function or both (depending of the cell signalling context – grey); (**b**) *SETD7 function according to cancer type.* Cellular processes modulated by SETD7 in each cancer type were grouped into tumour-promoting (red) or tumour-suppressing (green) or both (depending of the context – grey). The y axis shows the number of published papers describing SETD7 function included in this review; (**c**) *Type of dataset used to study SETD7 mRNA and protein expression in human cancer samples.* Studies were divided according to the source of mRNA and protein data used. The number of published papers in relation to the type of cancer is shown. Note that in some cases the same paper was considered more than once if it analysed SETD7 expression by more than one approach.
