# Supplementary material for: A Systematic Review to Define the Multi-Faceted Role of Lysine Methyltransferase SETD7 in Cancer
Source: Cancers (Basel). 2022 Mar 10;14(6):1414. doi: 10.3390/cancers14061414 (PMC8946661; doi:10.3390/cancers14061414)
Supplement: Supplementary file 1 [file cancers-14-01414-s001.zip › SuppTable S1.pdf]

Supplementary Table S1. Studies excluded after full text analysis and motives. Underlined studies were mentioned in the main text when appropriate, and their findings were integrated into the pathway analysis.

PMID	Exclusion criteria
18650421	diabetes and inflammation, not cancer
20121546	gallbladder cancer, SETD7 was downregulated by silencing T-Cadherin. No functional nor association studies regarding SETD7 or SETD7-induced methylation
21115810	SETD7 methyltates MYPT1, no functional nor association studies regarding SETD7 or SETD7-induced methylation in cencer cells or tissues, methods poorly described
27913949	prostate, no functional nor association studies regarding SETD7or SETD7-induced methylation
26861389	it doens't focus neither on SETD7 nor cancer. SETD7 was found to be related with serum lycopene concentrations in a healthy group of amish people
25189356	SETD7 was found mutated in prostate cancer. No functional nor further studies were conducted
30442713	SETD7 methylates SOX2, however, no functional nor association studies regarding SETD7or SETD7-induced methylation were performed. Mouse Embryonic Fibroblasts, HEK293, ovarian cancer cells PA-1
29358331	SETD7 methylates SOX2. No functional nor association studies regarding SETD7or SETD7-induced methylation, however they assess cells growth by looking at the cells under microscope. Mouse Embryonic Fibroblasts, HEK293, ovarian cancer cells PA-1
28534506	SETD7 methylates HIf1a, no functional nor association studies regarding SETD7 or SETD7-induced methylation were performed in cancer cells or tissues
25681344	SETD7 in Hepatitis C virus (HCV) infection, Huh7.5.1, Huh7, and L02 (normal human hepatic cell line), no functional studies
24492612	SETD7 methylates Dnmt1, no functional nor association studies regarding SETD7 or SETD7-induced methylation were performed
22820736	SETD7 methylates FOXO3. No functional nor association studies regarding SETD7 or SETD7-induced methylation
22007908	no functional nor association studies regarding SETD7 or SETD7-induced methylation
21440447	SETD7 inhibitors, no functional nor association studies regarding SETD7 or SETD7-induced methylation
19864627	SETD7 methylates p65 and is required for the expression of a subset of NF-kappaB target genes in response to TNFalpha stimulation. No functional nor association studies regarding SETD7 or SETD7-induced methylation, HeLa cells
18471979	SETD7 methylates ERa, no functional nor association studies regarding SETD7 or SETD7-induced methylation were performed
17108971	SETD7-mediated methylation of p53 K372 inhibits Smyd2-mediated methylation of p53 K370. No functional nor association studies regarding SETD7 or SETD7-induced methylation

15525938	SETD7 methylates p53 positively affecting its stability and restricting it to the nucleus. No functional nor association studies regarding SETD7 or SETD7-induced methylation
32126499	SETD7 inhibitors, no studies performed in cells
29869845	SETD7 inhibitors, no studies performed in cells
28389916	technique development, SETD7 inhibitors, no cells used
24650704	SETD7 inhibitors, no *in vitro* nor *in vivo* studies, no functional assays nor association analysis
32173197	focus on SETD7 inhibitors, cell proliferation assay used to test inhibitor's bilogical function in MCF7 breast cancer cells, but no controls were used
29498708	SETD7 inhibitors, cell proliferation assay using MCF7 (breast cancer), HL60 (human leukemia cell line), MV4-11 (human biphenotypic B myelomonocytic leukemia), Kasumi-1 (leukemic cell line), U937 (human myeloid leukaemia cell line), THP1 (human monocytic cell line derived from an acute monocytic leukemia patient) and Jurkat (human T lymphocyte cells that are used to study acute T cell leukemia) cell lines, no controls!!
21855805	p53 is methylated by SETD7, not cancer cells, *in vivo* experiments and *in vitro* using mouse embryonic fibroblasts
18280244	p53 is methylated by SETD7, not cancer cells, i*n vivo* experiments and *in vitro* using mouse embryonic fibroblasts
31035088	airway smooth muscle, functional studies (proliferation and invasion) but not cancer, primary cell culture from mice, mouse embryonic fibroblasts
30674889	problematic cell line used! focus molecule of the study isn't SETD7. SETD7 acts as a co-factor of ISL1 and links to ZEB1 *in vitro* (ChIP)
30396921	no functional nor association studies regarding SETD7 or SETD7-induced methylation, however SETD7 is the focus; SETD7 knockdown affects a large number of genes expression in LNCaP cell line
28557056	not cancer, SETD7 is the focus, *in vivo* experiments in mice, NCTC 2472 cells, Cyproheptadine (10-80 nmol, 1-6h) used as SETD7 antagonist
33305533	not cancer (myoblasts)
