# Supplementary material for: A Systematic Review to Define the Multi-Faceted Role of Lysine Methyltransferase SETD7 in Cancer
Source: Cancers (Basel). 2022 Mar 10;14(6):1414. doi: 10.3390/cancers14061414 (PMC8946661; doi:10.3390/cancers14061414)
Supplement: Supplementary file 1 [file cancers-14-01414-s001.zip › SuppTable S2.pdf]

Supplementary Table S2. Summary of the characteristics of each study.

Study	Type of Cancer	Cell cultures	Knock-down/ Over-expression of SETD7 or SETD7-methylated proteins (time of transfection)	SETD7 Inhibitor (concentration, time)	*In vivo* studies	*In vitro* Functional studies	Other assays	SETD7 antibody	Tumour-promoting/ suppressing role	Public online database(s)	Own human patient cohort (analysis technique)
			**Functional studies**							**Expression studies**	
Zipin-Roitman A et al., 2017 [33]	Acute myeloid leukemia	TEX, OCI-AML2, AML-193, Jurkat cells and Primary AML and chronic myelogenous leukemia cells	NA	(R)-PFI-2 (10uM)	NA	Trypan Blue	NA	not specified	Tumour-promoting	NA	NA
Gu Y et al., 2017 [31]	Acute myeloid leukemia and Lung Cancer	NB4, HEL, HL-60, KG-1, KG-1a, THP-1, H1299, A-549	Over-expression and knock-down by shRNAs (48h)	NA	NA	Annexin V/PI, Hoechst 33342 staining	NA	2813, Cell Signaling	Tumour-promoting/ -suppressoring	NA	Bone marrow aspirates from 35 AML patients and 8 healthy donors (RT-PCR)
Xie H et al., 2020 [34]	Bladder cancer	T24, UM-UC-3, SV-HUC-1	Over-expression and knock-down by siRNAs (48-72h)	NA	NA	Transwell assay, wound healing assay	IP	ab14820, Abcam	Tumour-suppressing	TCGA	NA
Song Y et al., 2016 [40]	Breast cancer	MCF-10A, BT-549, MDA-MB-468, MDA-MB-231, MCF-7	Over-expression and knock-down by shRNAs (2 weeks selection)	NA	NA	Transwell assay, MTT, colony formation assay, IF ki67	NA	Santa Cruz Biotech	Tumour-suppressing	NA	79 normal breast and 79 BC tissues (RT-PCR)

Si W et al., 2020 [45]	Breast cancer	MCF-7, MDA-MB-231, MDA-MB-468, T-47D, UACC-812, MCF-10A	Knock-down by shRNA	NA	NA	Transwell assay, cck-8, colony formation assay	Co-IP, ChIP-seq	ab14820, abcam	Tumour-promoting	Human Protein Atlas, GEO (GSE9893 and GSE12276) and Kaplan Mayer plotter	NA
Huang R et al., 2017 [44]	Breast cancer	MDA-MB-231, MCF-7, HEK293T	Knock-down by shRNAs (48h)	NA	NA	cck-8, Annexin V, GSH and GSSG Assay and Reactive Oxygen Species Assay Kit	Dual-luciferase assay	Proteintech	Tumour-promoting	TCGA	NA
Zhang Y et al., 2016 [43]	Breast cancer	ZR-75-1, MCF-7, MDA-MB-231, HUVEC, HEK293T	Knock-down by shRNA (2 months selection) or siRNA (48h)	NA	Xenograft model: Tumor size measured using calipers and Tumor volume calculated	Chick chorioallantoic membrane assay, Tube formation assay, Wound healing assay, cell proliferation assay (not specified)	human VEGF ELISA kit, Luciferase assays, co-IP, ChIP	SAB1306218, Sigma-Aldrich	Tumour-promoting	NA	80 BC patients (IHC)
Montenegro MF et al., 2016 [36]	Breast cancer	MCF-7, MDA-MB-231	Knock-down by siRNA (24-48h)	(R)-PFI-2 (50uM, 3days)	Xenograft model: primary tumors and metastases analyzed using IVIS Imaging System	Wound healing assay, flow citometry to access stemness markers, colorimetric assay for mitochondrial function using XTT	ChIP	ab124708, Abcam	Tumour-suppressing	NA	77 BC samples and 10 benign tumours (RT-PCR, WB and IHC)
Takemoto Y et al., 2016 [21]	Breast cancer	MCF-7	Knock-down by siRNA (48h)	cyproheptadine	Xenograft model: Tumor diameters measured with a caliper and tumor volume calculated	Cell counting	NA	Rabbit polyclonal, Cell Signaling Technology	Tumour-promoting	NA	NA
Shen C et al., 2015 [48]	Cervical cancer	HeLa, HEK293T	Knock-down by siRNA (48h) or shRNAs	NA	NA	count clones by crystal violet	IP	Upstate Biotechnology	Tumour-suppressing	NA	NA

Wu XN et al., 2017 [49]	Cervical cancer	HeLa, HEK293T	Knock-out of SETD7, YY2 or LSD1 by CRISPR/cas9 and Over-expression of YY2 or YY2 (K247R) (48h)	NA	Xenograft model: Tumor weight assessment	cell counting and colony formation assay	ChIP, ChIP-seq	07–314, Upstate	Tumour-suppressing	NA	NA
Hong X et al., 2018 [53]	Colorectal and gastric cancers	LOVO, RKO, HCT 116, SW480, HEK293T, MKN45, MEFs and a normal human intestinal epithelial cells	Knock-down by shRNA (2 weeks selection); RioK1-K411R and K413R mutants were introduced using the QuikChange site-directed mutagenesis kit	NA	Xenograft model: Tumor volume	Transwell assay, wound healing assay, CCK-8 and MTT	IP	homemade rabbit anti-SETD7 polyclonal antibody was raised against recombinant GST-SETD7 protein and mouse anti-SETD7 monoclonal antibodies from Millipore and Cell Signaling	Tumour-suppressing	NA	104 pairs of CRC samples (IHC)
Duan B et al., 2018 [42]	Colorectal cancer	HCT 116, RKO	Knock-down by siRNA (24-48h)	NA	NA	MTT, PI, Annexin V	NA	24840, Proteintech	Tumour-promoting	TCGA and Geo (GSEs not specified) for CRC, GC, HCC, PTC, LCa and BC	serum samples from 115 paired pre- and post-operative CRC, 38 colorectal polyps, and 38 healthy control patients (ELISA) 176 CRC and 20 colorectal polyps and para-cancerous tissue (IHC)
Wang LQ et al., 2018 [54]	Colorectal cancer	HCT 116, HEK293FT	Knock-down by siRNA (48h)	NA	NA	Flow citometry to access stemness markers	NA	Cell Signaling Technology	Tumour-suppressing	TCGA	NA

Zhang SL et al., 2020 [55]	Colorectal cancer	HCT 116, SW480	Over-expression and knock-down by siRNA (48h)	NA	NA	Transwell assay, wound healing assay, cck-8	Co-IP	NB100-56664SS, Sigma	Tumour-suppressing	NA	54 CRC and adjacent tissues (IHC)
Vasileva E et al., 2020 [6]	Colorectal cancer	HCT 116, HEK293T	Knock-out by CRISPR/cas9	NA	NA	PI, Annexin V	IP	2813s, Cell Signaling	Tumour-suppressing	GEO (GSE39582)	NA
Liu Z et al., 2019 [51]	Colorectal cancer	HCT 116, Co-115, SW48	Knock-down by shRNAs (48h)	Resveratrol (10-50uM, 24h)	NA	CellTiter-Glo Luminescent cell viability assay	Luciferase assay	2813	Tumour-suppressing	GEO (GSE17537: 244653_at)	NA
Francis NJ et al, 2012 [18]	Colorectal cancer	HCT 116	NA	610930-N (52-104 µM, 24-48h)	NA	sulforhodamine B	ELISA assay	NA	Tumour-promoting	NA	NA
Xie Q et al., 2011 [50]	Colorectal cancer and Osteosarcoma	U2OS, HCT 116	Over-expression and knock-down by siRNA (36-48h)	NA	NA	Colony formation assay, Annexin V	Luciferase assay	Not specified	Tumour-suppressing	NA	NA
Zhou J et al., 2015 [56]	Epithelial ovarian cancer	OVCAR-3, SKOV3, NOE095, hOSEpiC	Over-expression and knock-down by siRNAs (48h)	NA	NA	Transwell assay, wound healing assay, count clones by crystal violet	Luciferase assay	mouse, Abcam	Tumour-promoting	NA	60 EOC and their adjacent normal tissues (qPCR)
Akiyama Y et al., 2016 [57]	Gastric cancer	AGS, GCIY, HSC-43, HSC-44PE, HSC-57, HSC-60, NUGC-4, KATO-III, MKN7, MKN45, MKN74, TGBC11TKB	Knock-down by siRNA (48-72h)	NA	NA	Transwell assay, cck-8	qChIP	Mouse monoclonal,clone 5F2.3, 04-0805, Merck Millipore	Tumour-suppressing	NA	376 primary GCs (IHC)
Li C et al., 2020 [58]	Glioma	HEB and U251	Knock-down by siRNA	NA	NA	Cck-8, transwell assay	ChIP, Luciferase assay	NA	Tumour-suppressing	NA	20 pairs of glioma tissue samples and adjacent non-tumorous tissues (RT-qPCR)

Wang H et al., 2021 [59]	Hepatocellular carcinoma	Hep3B, Huh7, HCCLM3	Over-expression by SETD7 expressing vectors and knock-down by siRNA (48h)	NA	NA	Cck-8, EdU assay, transwell assay and wound healing assay	RNA IP	Mouse monoclonal, ab14820, Abcam	Tumour-suppressing	NA	100 primary HCC (complicating with PVTT, n = 15; without PVTT, n = 85), 100 corresponding peri-tumour tissues and 10 normal hepatic samples (RT-qPCR)
Gu Y et al., 2018 [32]	Hepatocellular carcinoma	Huh7	Knock-down by shRNA (48h)	MTA (1mM, 24h)	NA	Transwell assay, cck-8	Co-IP	2813, Cell Signaling Technology	Tumour-promoting	TCGA: CRC, Head and neck, kidney, LCa, PCa, PTC, uveal; Oncomine: CRC, kidney, AML, BC	68 HCC and matched adjacent healthy tissues (IHC)
Chen Y et al., 2016 [60]	Hepatocellular carcinoma	NA	NA	NA	NA	NA	NA	NA	Tumour-promoting	NA	20 pairs of HCC and adjacent non-tumorous tissues (qRT-PCR and WB) 225 pairs of HCC and adjacent non-tumorous tissues (IHC)
Kim Y et al., 2016 [83]	Lung cancer and Breast cancer	HEK293T, HeLa, MDA-MB231, MEFs, Lewis lung carcinoma cells	Knock-in mice bearing a methylation-defective *Hif1a*$^{KA/KA}$ allele	NA	Mice and xenograft models: Tumour volume measured with a calliper and tumour weight	scratch-cell motility assay, transwell assay, colony formation assay, IF of CD31 (vascularization), ki67	IP	2813, Cell Signalling	Tumour-suppressing	NA	NA

						(proliferation), caspase 3 (apoptosis)					
Cao L et al., 2020 [64]	Lung cancer	A-549, H1299, H661, BEAS-2B	Over-expression and knock-down by siRNAs (24-72h)	NA	NA	Transwell assay, wound healing assay, cck-8	NA	ab14820	Tumour-suppressing	NA	10 pairs of LCa and matched non-tumour tissues (qPCR and WB)
Meng F., 2020 [67]	Lung cancer	16HBE, A-549, PC-9, HCC827, NCI-H1975 and H1299	Knock-down by shRNAs (48h)	NA	NA	Colony formation assay, EdU assay, transwell assay, TUNEL assay and flow cytometry	RNA IP, Luciferase assay	Abcam	Tumour-promoting	NA	64 pairs of LCa and adjacent non-tumour tissues (RT-qPCR)
Daks A et al., 2021 [63]	Lung Cancer	A-549, H1299 and patient-derived cells	Over-expression and knock-down by shRNA and knock-out by CRISPR/cas9	(R)-PFI-2 (2uM, 48h)	NA	Real-time cell index monitorization (xCELLigence), cell migration assay (CIM-plate), colony formation assay, MTT, PI, Annexin V	NA	Cell signalling	Tumour-promoting / Tumour-suppressing	GEO (GSE11969)	NA
Wang D et al., 2013 [62]	Lung cancer	HEK293T, H1299, MEFs	Knock-down by siRNA (48h)	NA	NA	count clones by crystal violet	Luciferase assay, co-IP	Millipore, Cell Signalling Technology	Tumour-suppressing	NA	NA
Fu L et al., 2016 [66]	Lung cancer	HEK293T, A-549, NIH-3T3, BEAS-2B	Knock-down by siRNA (24h)	NA	Xenograft model: Tumour size and volume	Wound healing assay, MTT	Luciferase assay, co-IP, ChIP	Mouse, Cell signalling	Tumour-promoting	NA	NA
Lezina L et al., 2014 [65]	Lung cancer and osteosarcoma	H1299, H23, H520, H522, H1650, H460, U2OS, HEK-293	Knock-down by shRNA	NA	NA	PI, BrDU, IF of anti-phospho-Histone H3 (Ser10) (mitotic marker)	Luciferase assay, ChIP	not specified	Tumour-promoting	GEO: 2 LCa datasets (GSE31210 and GSE36471)	NA
Lezina L et al., 2015 [41]	Lung cancer and osteosarcoma	H522, H1650, H1299, U2OS	Knock-down by siRNA (48h)	NA	NA	DNA damage analysis using reporter constructs; IF of γ-H2Ax; comet assay,	NA	Cell Signalling	Tumour-promoting	GEO: BC dataset (GSE22226)	NA

						colony formation assay, PI					
Kontaki H et al., 2010 [38]	Lung cancer and osteosarcoma	H1299, SaOS-2	Over-expression and knock-down by shRNAs	NA	NA	MTT, PI, PARP cleavage and caspase3/7 activity assays	ChIP	07-314, Millipore	Tumour-promoting	NA	NA
Carr SM et al., 2011 [72]	Osteosarcoma	U2OS, HeLa, SaOS-2	Over-expression of pRb K810R; SETD7 Knock-down by siRNA (20nM, 72h)	NA	NA	PI	Luciferase assay, IP, ChIP	polyclonal, Cell signalling	Tumour-suppressing	NA	NA
Munro S et al., 2010 [71]	Osteosarcoma	CC42, C2C12, U2OS, SaOS-2	Over-expression of pRb K873A; SETD7 Knock-down by siRNA (25nM, 24-72h)	NA	NA	PI	Luciferase assay, ChIP	polyclonal, Millipore	Tumour-suppressing	NA	NA
Ivanov GS et al., 2007 [52]	Osteosarcoma	U2OS, HEK293, H1299	Knock-down by shRNA	NA	NA	PI	IP	not specified	Tumour-suppressing	NA	NA
Liu Q et al., 2015 [74]	Osteosarcoma	U2OS	Knock-down by shRNA and siRNAs	NA	NA	Cell counting	Luciferase assay, co-IP, ChIP	Millipore	Tumour-promoting	NA	NA
Zhao M et al., 2020 [81]	Papillary thyroid carcinoma	TPC-1, B-CPAP	Over-expression and knock-down by shRNAs (48h)	NA	NA	cck-8, colony formation assay, PI, Annexin V	Luciferase assay	rabbit, Abcam	Tumour-promoting	NA	15 PTC and neighboring normal tissues<br><br>(qPCR)
Song H et al., 2020 [75]	Prostate Cancer	LNCaP, PC-3, HEK293T	Over-expression of RORα2 K87R; Induction of SETD7 by transfecting the cells with Hismax-SETD7	NA	NA	CellTiter 96®AQueous One Solution Reagent	Luciferase assay, ChIP	not specified	Tumour-promoting	NA	NA
Wang C et al., 2018 [76]	Prostate Cancer	LNCaP, PC-3	Knock-down by shRNA	Phenethyl isothiocyanate (PEITC - 5 μM, 24h) and ursolic	NA	comet assay, colony formation assay, Intracellular ROS measurement by FACS of CM-	NA	Cell Signaling Technology	Tumour-promoting	NA	NA

				acid (UA - 20 µM, 24h)		H2DCFDA stained cells					
Gaughan L et al, 2011 [79]	Prostate Cancer	HEK293T, LNCaP, PC-3	knock-down by shRNA (2 weeks selection) and knock-down by siRNA (72h)	NA	NA	WST-1, caspase-3 expression analysis	NA	clone 5F2.3, Millipore	Tumour-promoting	NA	76 PCa and 24 benign prostate tissues (IHC)
Oudhoff MJ et al., 2016 [8]	Small-intestine cancer	NA	Mice knock-out; knock-down by siRNA for cells	(S) and (R)-PFI-2 (1-10uM, 30min-4h)	Mice model: Number of tumour, lifespan assessment	Organoid formation	ELISA, IP	2D10, Abcam	Tumour-promoting	NA	NA

BC – breast cancer; ChIP – chromatin immunoprecipitation; CRC – colorectal cancer; HCC – hepatocellular carcinoma; IHC – immunohistochemistry; IP – immunoprecipitation; MEFs – mouse embryonic fibroblasts; MTT – 3-(4,5-dimethylthiazol-2-yl)-2,5-diphenyltetrazolium bromide; NA – not applicable; PI – propidium iodide; RT-PCR – reverse transcriptase polymerase chain reaction; PTC – papillary thyroid cancer; PVTT – portal vein tumour thrombus; WB – Western blot; XTT – sodium 3´-[1- (phenylaminocarbonyl)- 3,4- tetrazolium]-bis (4-methoxy6-nitro) benzene sulfonic acid hydrate
